# The Transition of Mg-Containing Phases and Recovery of NaCl in Molten Salt Chloride Slags at High Temperature

**DOI:** 10.3390/ma15175983

**Published:** 2022-08-30

**Authors:** Feng Chen, Yuekai Wen, Yufeng Guo, Shuai Wang, Lingzhi Yang, Yu Zheng, Dongyue Li, Yuqiao Ren

**Affiliations:** School of Minerals Processing and Bioengineering, Central South University, Changsha 410083, China

**Keywords:** molten salt chloride slags, phases transition of Mg-containing phases, removal rate of Mg, recovery rate of NaCl

## Abstract

The molten salt chlorination method is more suitable to produce TiCl_4_ using titanium-containing materials with high contents of CaO and MgO in China. However, there is a large amount of molten salt chloride slags generated from the molten salt chlorination process, which contains a variety of chlorides and is difficult to treat, often causing serious environmental problems such as direct piling or landfilling. A novel process was proposed to deal with molten salt chloride slags, and calcium chloride could be effectively removed by this process (as in our previous study). However, magnesium chloride is another impurity which can deteriorate the density and viscosity of the molten salt; it is often found in higher contents, and should be also removed from molten salt chloride slags to efficiently recycle NaCl in novel processes. Na_2_SiO_3_ is added to the molten salt chloride slags in the molten state to produce the Mg-containing solid phase, which could be separated with the molten NaCl in novel processes. Thus, the transition of Mg-containing phases and the recovery of NaCl in molten salt chloride slags at high temperature in a novel process were systematically investigated in this work, including thermodynamic analysis, the phase transition behavior of Mg-containing phases, NaCl recovery, etc. The removal rate of Mg was 99.56% when the molar ratio of MgCl_2_:Na_2_SiO_3_ was 1:1.5 at 1273 K and in a N_2_ atmosphere. The recovery rate of NaCl from the molten salt chlorination slag was 97.62% and the purity of NaCl obtained was 99.35 wt%, which could be used in the molten salt chlorination process.

## 1. Introduction

China’s Panxi region is rich in titanium reserves, mainly distributed in vanadium and titanium magnetite [1,2]. Titanium resource accounts for 35% of the world’s primary ilmenite reserves and 93% of China’s primary ilmenite reserves [3,4,5]. However, the grade of titanium ore is relatively low and the content of calcium and magnesium impurities is high [6,7]. TiO_2_ content in titanium concentrate is only between 46% and 48% [8,9].

TiCl4, a key intermediate raw material in the preparation of titanium products, is mainly prepared by boiling and molten salt chlorination. However, the boiling chlorination process requires a high level of titanium raw material. This low-grade titanium ore with high calcium and magnesium content cannot be used in the boiling chlorination process to produce TiCl_4_ [10,11].

Titanium resources with high calcium and magnesium content are more applicable for TiCl_4_ production using molten salt chlorination technology [12,13,14]. However, the biggest problem of the molten salt chlorination process is the large amount of molten salt chloride slags generated [15,16]. When producing 1 t of titanium tetrachloride, about 200–500 kg of molten salt chloride slag can be generated [17]. These slags often accumulate in large quantities and are not effectively utilized, causing serious environmental pollution [18]. The disposal of molten salt chloride slags has become a serious problem in the molten salt chlorination process [19].

Currently, there is little research concerning the treatment and utilization of molten salt chloride slags (neither domestic nor overseas). The main treatment methods used are the pile burial method and the water-soluble method. The pile burial method refers to burying the molten salt chloride slags in the waste mine or using lime intervals to place them on the wasteland. This method can cause environmental pollution. In the literature, scholars first dissolved molten salt chlorinated slag in water and then processed the dissolved solution and filtered slags to recover useful substances [20,21,22,23]. However, the water-soluble method is a complex process with high secondary waste generation and fails to fundamentally solve the problem of molten salt chloride slags [22,23]. Moreover, NaCl, the main component of molten salt chloride slags, is necessary for the process of molten salt chlorination. The question concerning how to recover NaCl from molten salt chloride slags reprsents the key to the recycling of molten salt chloride slags.

The composition of molten salt chloride slag is complex. Here, calcium and magnesium impurities mainly exist in the form of chloride salts, with a CaCl_2_ content of 8% and MgCl_2_ content of 18% [12]. Excessive magnesium chloride content will lead to higher density and viscosity of the molten salt [24,25,26]. This affects the surface wetting ability of solid particles in molten salt by influencing its fluidity and bubble rise rate, which is harmful to the molten salt chlorination process [27,28]. In this paper, a new process for treating molten salt chloride slag is proposed to remove MgCl_2_ from molten salt chloride slag by the high-temperature phase transition method. How to remove the MgCl_2_, the most abundant impurity, from molten salt chlorine slag represents the key issue of molten salt chlorine slag treatment.

In the available literature, scholars used the strongly oxidizing, alkaline waste brine from the purification of molten salt chlorination tail gas to treat the leachate of molten salt chloride slags and then recover NaCl and MgCl_2_ from it [29]. However, this method is a complicated process and has a low recovery efficiency. In this paper, a new process of high-temperature phase transition method was used to treat molten salt chloride slag. When Na_2_SiO_3_ was added to the molten salt chloride slags at a high temperature, NaCl remained in the liquid state to produce the Mg-containing solid phase. The liquid NaCl was separated from the Mg-containing solid phase and reused in the molten salt chlorination process. Solid magnesium-containing silicate could be used as a constructal material, so that all magnesium chloride and sodium chloride were recycled.

The transition of Mg-containing phases and their separation of NaCl are the key parts of this work, and they are systematically studied in this paper. Thermodynamic analysis, phase transition behavior, and Mg removal behavior are all investigated.

## 2. Materials and Methods

### 2.1. Materials

#### 2.1.1. Raw Materials

The main chemical reagents involved in the study of phase transition and impurity removal by pure reagents simulating molten salt chloride slags are sodium chloride, anhydrous magnesium chloride, and anhydrous sodium metasilicate. These are chemical analysis pure reagents. Their specific specifications are shown in Table 1.

**Figure 1:**
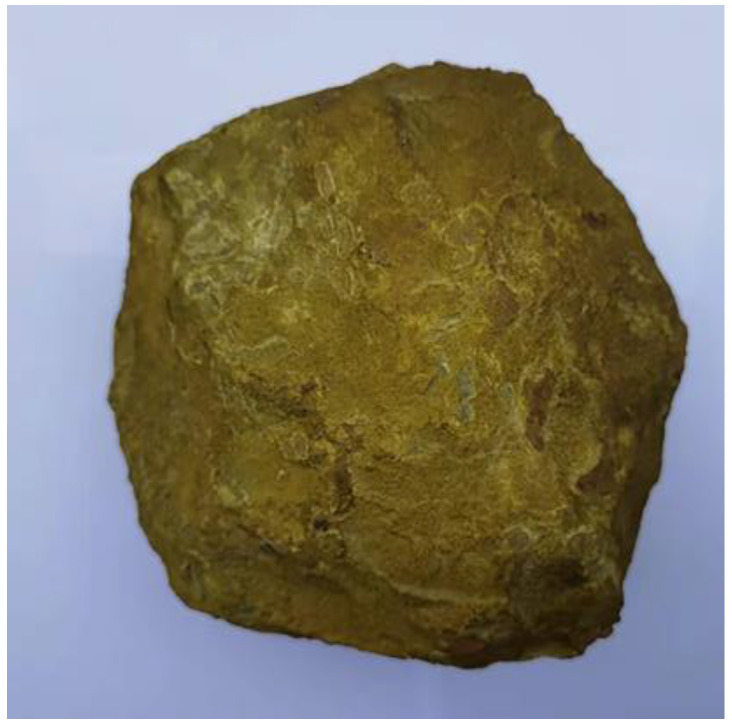
Molten salt chloride slags.

The molten salt chloride slags used in the study were from Panxi, as shown in Figure 1. The molten salt chloride slag has a dark greyish yellowish surface and is hard. The main chemical composition of the molten salt chloride slag is shown in Table 2.

The filter was a self-purchased zirconia honeycomb filter with a pore size of 30 ppi (approximately 0.8 mm). The zirconia honeycomb filter was consistent with the honeycomb filter used in our previous study [30].

#### 2.1.2. Experimental Systems

Three kinds of molten salt systems, including simulative slags, synthetic slags, and molten salt chloride slags from Panxi, were used in this experiment.

The weight ratio of NaCl:MgCl_2_ in the simulative slags is 9:1. The weight ratio of NaCl:MgCl_2_:CaCl_2_:C in the synthetic slags is 75:15:5:5. The 45g of raw material was weighed for each experiment. The weight of the additive (Na_2_SiO_3_) varied with the MgCl_2_ (and CaCl_2_) in the molten salt system.

### 2.2. Methods

#### 2.2.1. Chemical Composition Analysis

In this study, Ca, Mg, Al, Fe, and Cl were determined by titrimetric method following as relevant National testing standard. Among them, the detection of Mg is based on GB/T 21525-2008. Na and Mn were determined by atomic absorption. Si was determined by weight method in this study. Ti was determined by inductively coupled plasma spectrometry (ICP).

#### 2.2.2. Thermodynamic Analysis

Thermodynamic calculations of the MgCl_2_-Na_2_SiO_3_-NaCl system were carried out using Factsage 8.1. The calculation process used the “Phase Diagram” and “Reaction” modules. Thermodynamic calculations were used as a theoretical basis to guide the subsequent experiments.

#### 2.2.3. High Temperature Phase Transition and Filtration Experiment

**Figure 2:**
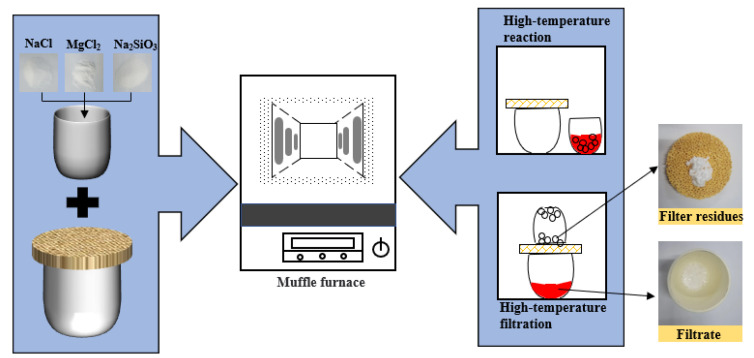
Schematic diagram of experiment.

The reagents used in the experiment should be dry and water-free. The reagents are placed in a corundum crucible and stirred thoroughly with a stirrer in a vacuum glove box. The stirrer was stirred at a rate of 200 r/min for 2min. As reagents tend to absorb water, the raw material must first be dried in a drying oven to a constant weight. The temperature of the drying oven is generally 383 ± 5 K. The experiments were carried out in a high-temperature muffle furnace. The raw material is reacted in a corundum crucible at a fixed temperature for 1 h. After the complete reaction of the substances, the separation of filtrate and filter residues is carried out by a filtering operation. The specific experimental procedure was basically the same as our previous study [30]. The filtrate after solidified and filter residues are shown in Figure 2.

#### 2.2.4. Analytical Method

(1) Phase analysis

The filtrate and filter residues were tested using X-ray diffraction analyzer (D8 Advance, Bruker, Saarbrucken, Germany) provided by the Analysis and Testing Center of the Central South Institute of Mining and Metallurgy. X-ray diffraction angles (2θ) are 10° to 90° and the scanning time is 30 min. The XRD pattern processing Materials Data jade software (Jade 6.5, Beijing, China) was used for the physical phase retrieval and analysis.

(2) Mg removal rate

The purpose of the high-temperature solid-phase transition of MgCl_2_ using Na_2_SiO_3_ as an additive is to separate Mg from the molten salt chloride slags. The study focused on the solution obtained after solid-liquid separation of the reaction products. The content of Mg impurity elements was analyzed. The removal rate of Mg was calculated before and after the high temperature reaction. The Mg removal rate is calculated in Equation (1) and the parameters are shown in Table 3.

(1) $$\eta_{\mathrm{Mg}}=\left(1-\frac{m_{1}\times \omega_{\mathrm{Mg}}^{\prime }}{m\times \omega_{\mathrm{Mg}}}\right)\times 100\%$$

(3) Recovery rate of NaCl

Na_2_SiO_3_ is added to the molten salt chloride slags in the molten state to produce the Mg-containing solid phase, which could be separated with the molten NaCl in the novel process. This will result in a higher purity of the resulting NaCl molten salt. The recovery rate of NaCl after high temperature separation was calculated. The calculation formula of NaCl recovery is shown in Equation (2), and the parameters are shown in Table 3.

(2) $$P_{\mathrm{NaCl}}=\frac{m_{1}\times \omega_{\mathrm{NaCl}}^{\prime }}{m\times \omega_{\mathrm{NaCl}}+m_{2}}\times 100\%$$

## 3. Thermodynamic Analysis

The reaction of MgCl_2_ with additives was analyzed by Gibbs free energy. Thermodynamic analysis was used to determine whether Na_2_SiO_3_ was a suitable additive. The reaction temperature was determined by analyzing the phase diagram of MgCl_2_-Na_2_SiO_3_-nacl system.

### 3.1. Thermodynamic Reactions of Phase Transition of MgCl_2_ at High Temperatures

**Figure 3:**
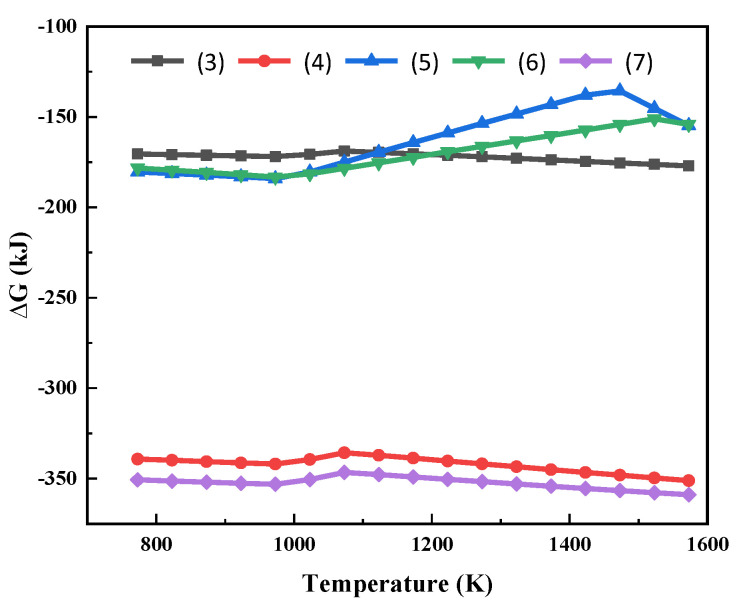
Gibbs free energy of the reactions (3)~(7) at different temperatures.

The possible chemical reactions between MgCl_2_ and Na_2_SiO_3_ (Additive) were analysed—Equations (3)–(7). The Gibbs free energy of the reaction at different temperatures is shown in Figure 3.

(3) $${\mathrm{MgCl}}_{2}+{\mathrm{Na}}_{2}{\mathrm{SiO}}_{3}={\mathrm{MgSiO}}_{3}+2\mathrm{NaCl}$$

(4) $$2{\mathrm{MgCl}}_{2}+2{\mathrm{Na}}_{2}{\mathrm{SiO}}_{3}={\mathrm{Mg}}_{2}{\mathrm{SiO}}_{4}+4\mathrm{NaCl}+{\mathrm{SiO}}_{2}$$

(5) $$3{\mathrm{MgCl}}_{2}+{\mathrm{Na}}_{2}{\mathrm{SiO}}_{3}={\mathrm{MgSiO}}_{3}+2{\mathrm{NaMgCl}}_{3}$$

(6) $$2{\mathrm{MgCl}}_{2}+{\mathrm{Na}}_{2}{\mathrm{SiO}}_{3}={\mathrm{MgSiO}}_{3}+{\mathrm{Na}}_{2}{\mathrm{MgCl}}_{4}$$

(7) $$2{\mathrm{MgCl}}_{2}+3{\mathrm{Na}}_{2}{\mathrm{SiO}}_{3}={\mathrm{Na}}_{2}{\mathrm{Mg}}_{2}{\mathrm{Si}}_{2}O_{7}+4\mathrm{NaCl}+{\mathrm{SiO}}_{2}$$

As can be seen from Figure 3, the Gibbs free energies for the reactions of MgCl_2_ with Na_2_SiO_3_ are all less than 0. This shows that the high temperature phase transition of Mg in the molten salt system can be realized thermodynamically. The reactions (3) to (7) can all occur within the temperature range in Figure 3. After the reaction, Mg is present in the form of silicate. At the same time, molten NaCl is generated. MgCl_2_ reacts preferentially with Na_2_SiO_3_ at high temperatures to produce sodium-magnesium silicate and silicon dioxide.

### 3.2. Reaction Equilibrium Phase Diagram Analysis

**Figure 4:**
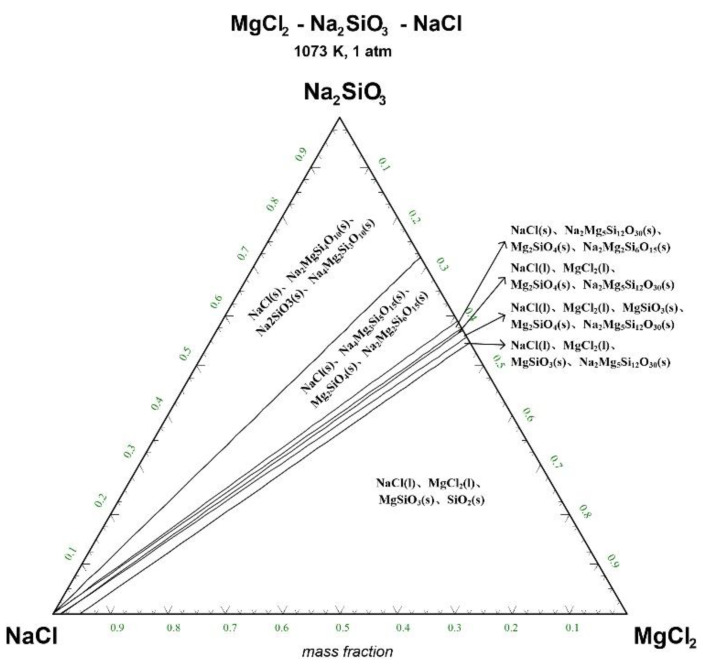
Ternary phase diagram of MgCl_2_-Na_2_SiO_3_-NaCl system at 1073 K. (l—liquid; s—solid).

**Figure 5:**
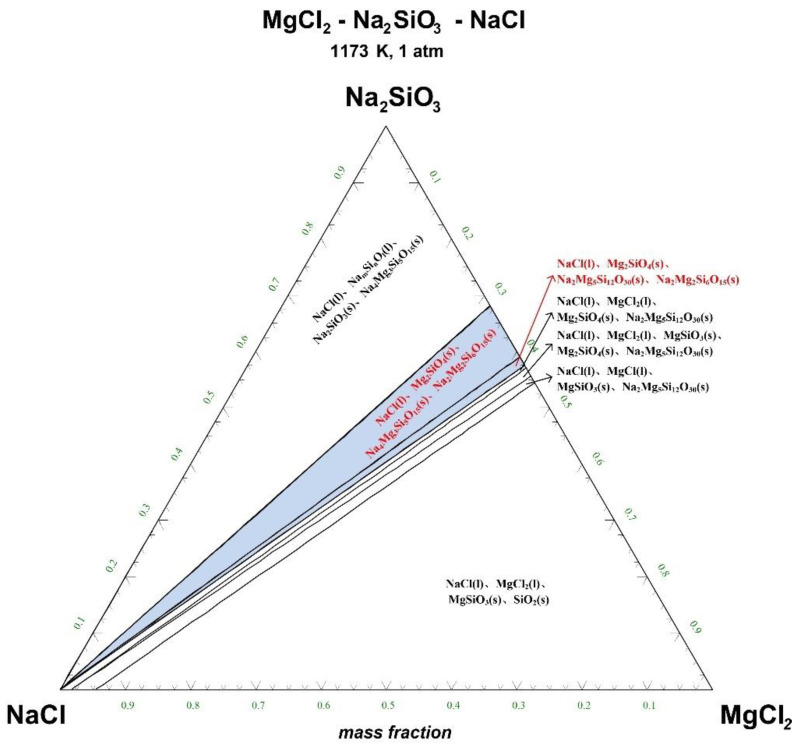
Ternary phase diagram of MgCl_2_-Na_2_SiO_3_-NaCl system at 1173 K. (l—liquid; s—solid).

**Figure 6:**
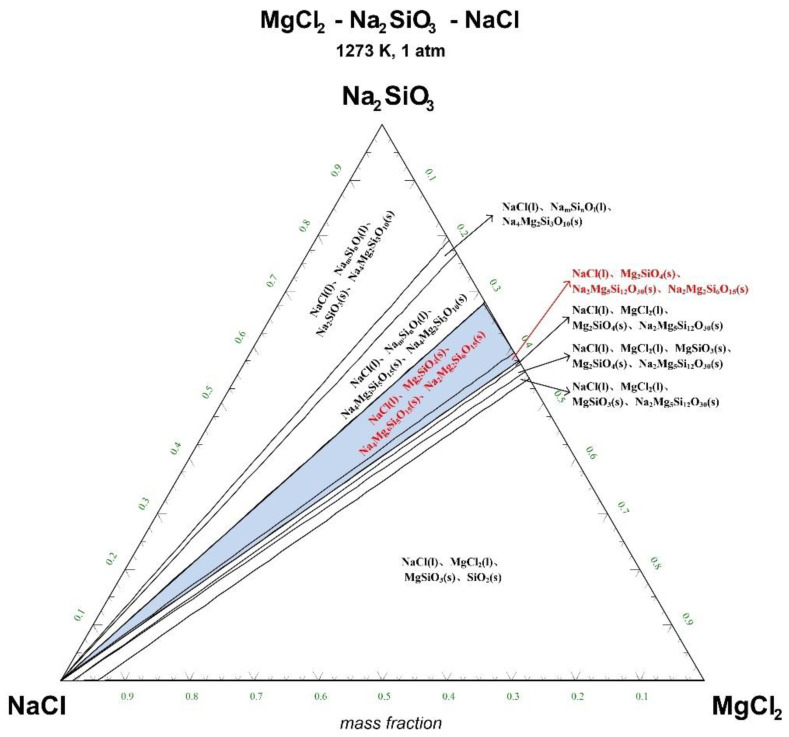
Ternary phase diagram of MgCl_2_-Na_2_SiO_3_-NaCl system at 1273 K. (l—liquid; s—solid).

**Figure 7:**
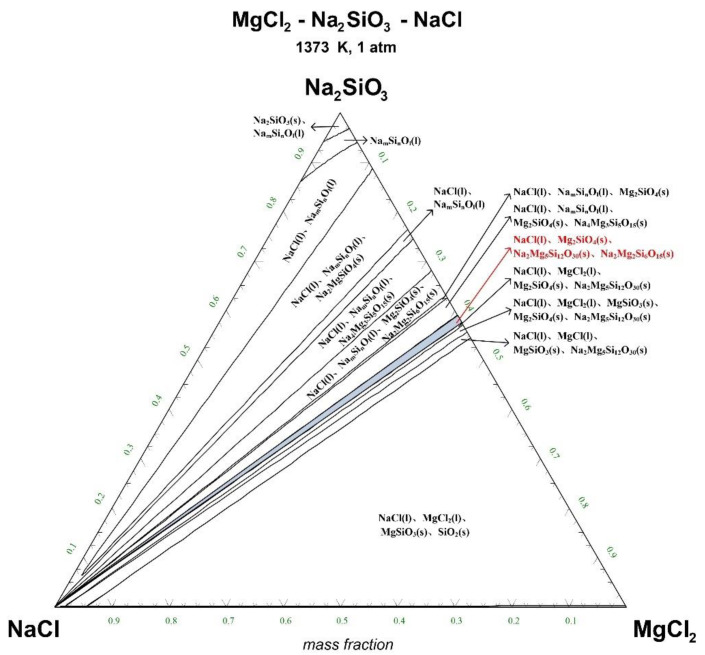
Ternary phase diagram of MgCl_2_-Na_2_SiO_3_-NaCl system at 1373 K. (l—liquid; s—solid).

The ternary phase diagrams of MgCl_2_-Na_2_SiO_3_-NaCl at a temperature of 1073 K, 1173 K, 1273 K, and 1373 K are shown in Figure 4, Figure 5, Figure 6 and Figure 7.

There are 10–20 wt% MgCl_2_ in typical molten salt chloride slags. When the temperature is 1073 K, liquid MgCl_2_ exists in the molten salt chloride slags, shown in Figure 4. It is difficult to separate MgCl_2_(l) from the liquid product to obtain NaCl(l) at this point. When the temperature rises to 1173 K, there exists a phase equilibrium state where only NaCl is the liquid product and the rest are sodium-magnesium silicate solid phase products, shown in the blue area of Figure 5. When the temperature rises to 1273 K and 1373 K, the reaction products of MgCl_2_ and Na_2_SiO_3_ are similar to the products produced at a temperature of 1073 K, shown in the blue area of Figure 6 and Figure 7.

The thermodynamic phase equilibrium shows that Na_2_SiO_3_ can react with molten salts containing Mg as an additive. When the temperature is higher than 1173 K, solid-liquid separation can be achieved under suitable conditions (blue area) to obtain the NaCl(l).

## 4. Phase Transition and Removal Behavior of Magnesium at High Temperature

Based on the reaction thermodynamics, the phase diagram of MgCl_2_, NaCl, and additive Na_2_SiO_3_ in salt chloride slag was analyzed in this study, and the pure reagent NaCl-MgCl_2_ was used to conduct high-temperature phase transformation experiment to simulate molten salt system. The high temperature solid phase transition and solid-liquid separation of MgCl_2_ were investigated by varying the experimental conditions. The effect of different experimental conditions on the removal of Mg^2+^ was investigated. The optimal conditions for impurity removal were determined experimentally.

### 4.1. Additive Dosage

**Figure 8:**
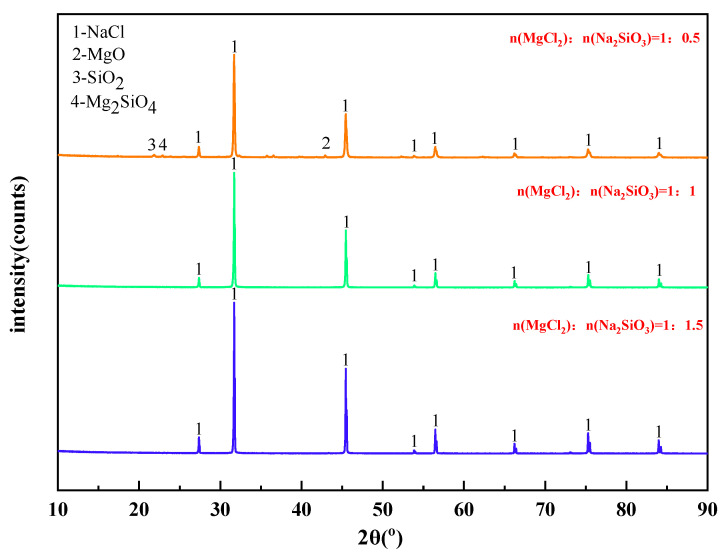
XRD of filtrate obtained with different dosage of additives.

The mass content of MgCl_2_ in raw materials is 10%. When the temperature was 1173 K, the X-ray diffraction pattern of filtrate obtained with different dosage of additives was shown in Figure 8. The effect of varying the amount of additives on the phase transition of the reaction products was analyzed.

It can be seen from Figure 8 that the main phase in the filtrate after high temperature filtration is NaCl with a small amount of MgO, SiO_2_, and Mg_2_SiO_4_ when the amount of additives is insufficient. However, when the additive dosage is doubled or tripled, only NaCl remains in the phase of the filtrate. Therefore, increasing the additive dosage can significantly improve the quality of the filtrate after high-temperature filtration.

**Figure 9:**
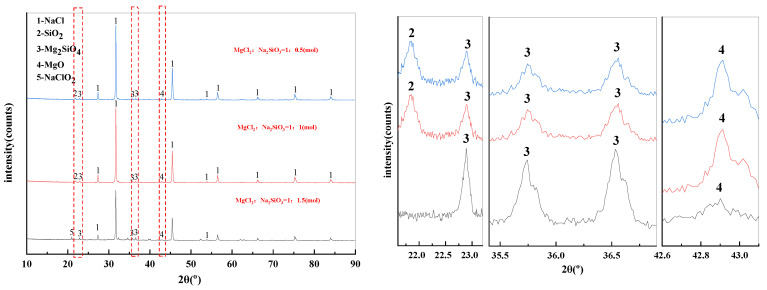
XRD of filter residues obtained with different dosage of additives.

The X-ray diffraction pattern of filter residues obtained with different dosage of additives was shown in Figure 9. When the MgCl_2_:Na_2_SiO_3_ molar ratio was 1:0.5 and 1:1, the substances contained in the filtered residues are NaCl, SiO_2_, Mg_2_SiO_4_ and MgO. When the additive dosage was increased to 1:1.5, the diffraction peaks of NaClO_2_ started to appear in the filtered residues.

**Figure 10:**
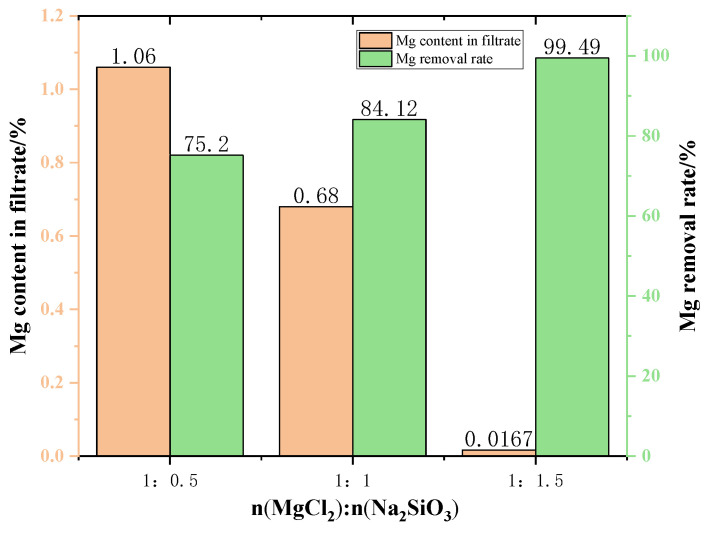
The effect of additive dosage on the Mg content in the filtrate and on the Mg removal rate.

Figure 10 shows the content of Mg in the filtrate at different additive dosages. The amount of Mg in the filtrate gradually decreases when increasing the amount of additives used in the experiment. This can be explained by the fact that the higher the amount of additive is conducive to the consumption of magnesium ions in the raw material, resulting in the reduction of magnesium ion content in the filtrate. When the MgCl_2_:Na_2_SiO_3_ molar ratio was 1:1.5, the filtrate obtained had the lowest Mg content and the quality of new molten salt was better.

The changes in the Mg removal rate of the filtered samples with different addition dosages are shown in Figure 7. The removal rate of Mg from the samples showed an increasing trend as the addition dosage increased. However, excessive additives will increase the impurities in the filtrate, resulting in lower quality of molten salt. Therefore, the appropriate dosage is the molar ratio of MgCl_2_:Na_2_SiO_3_ from 1:1 to 1:1.5.

### 4.2. Phase Transition Temperature

**Figure 11:**
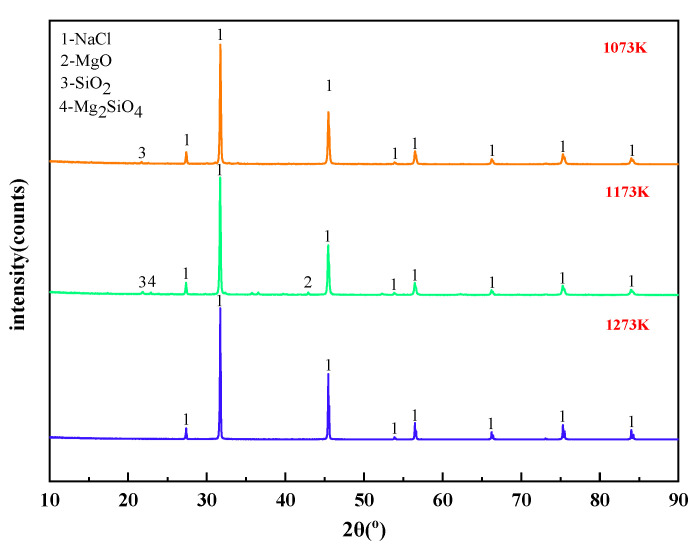
XRD of filtrate obtained with different temperatures.

When the molar ratio of MgCl_2_:Na_2_SiO_3_ was 1:0.5, the X-ray diffraction of the filtrate at different temperatures is shown in Figure 11. The effect of different temperature conditions on the phase change of the reaction products was analyzed.

It can be summarized from Figure 11 that the main substance in the filtrate obtained from the experiments under different temperature conditions was NaCl. This is consistent with the content of thermodynamic phase diagrams in Figure 4, Figure 5 and Figure 6. When the phase transition temperature was below 1273 K, the high-temperature phase transition reaction of Mg^2+^ did not proceed sufficiently. This results in some of the magnesium-silica phases remaining in the filtrate, which degrades the quality of the filtrate. When the phase transition temperature reaches 1273 K, only NaCl remains in the filtrate.

**Figure 12:**
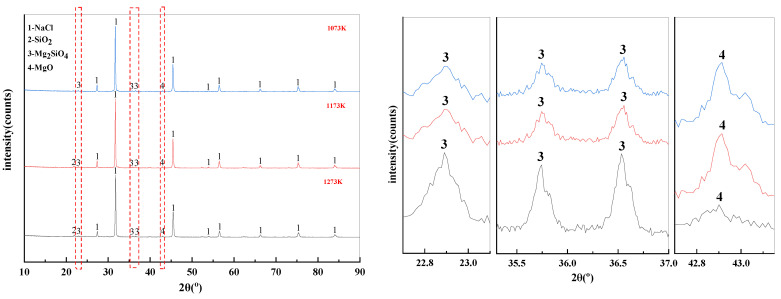
XRD of filter residues obtained with different temperature.

The effect of temperature on the phase transition behavior of filter residues are shown in Figure 12. There is also NaCl residue in the filter residues. In addition, MgCl_2_ reacts with Na_2_SiO_3_ at high temperature to form SiO_2_, Mg_2_SiO_4_, and MgO. According to the X-ray diffraction patterns of the filter residue at different temperatures, it is clear that increasing the temperature leads to a decomposition of the Mg_2_SiO_4_ produced by the reaction. This leads to an increase in the SiO_2_ and MgO content of the filter residues.

**Figure 13:**
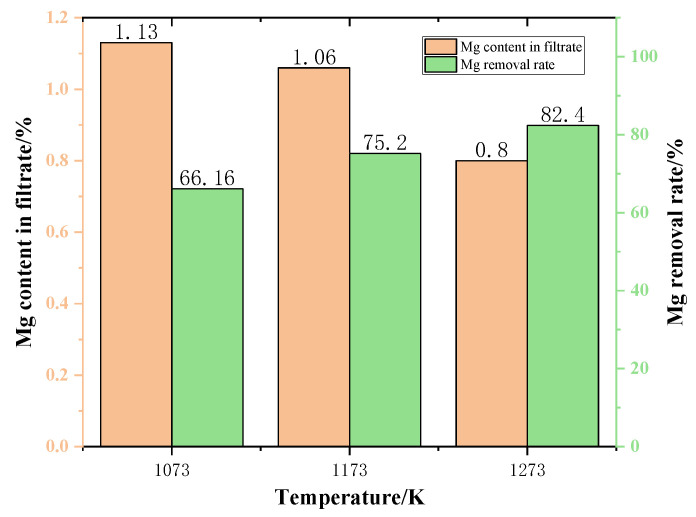
The effect of temperature on the content of Mg in the filtrate and on Mg removal rate.

Figure 13 shows the content of Mg in the filtrate at different temperatures. As the temperature increases, the Mg content in the filtrate gradually decreases. When the temperature was 1273 K, the Mg content dropped to 0.8%. As can be seen from Figure 13, the Mg content in the filtrate gradually decreased with the increase of the temperature. This is due to the fact that increasing the temperature is conducive to increasing the rate of reaction, thus reducing the content of Mg in the filtrate.

The variation of Mg removal rate of the samples after different temperature reactions is shown in Figure 13. The experimental results are shown in Figure 13. As the phase transition temperature increases, the removal of Mg also increases. This demonstrates that increasing the temperature is advantageous to the removal of Mg from the slags phase. When the phase transition temperature increased from 1073 K to 1273 K, the Mg removal rate increased by 24.55%. In a word, to maximize the Mg removal rate in the phase transition process and minimize the Mg content in the filtrate obtained by filtration, the temperature should be 1273 K.

### 4.3. Phase Transition Atmosphere

**Figure 14:**
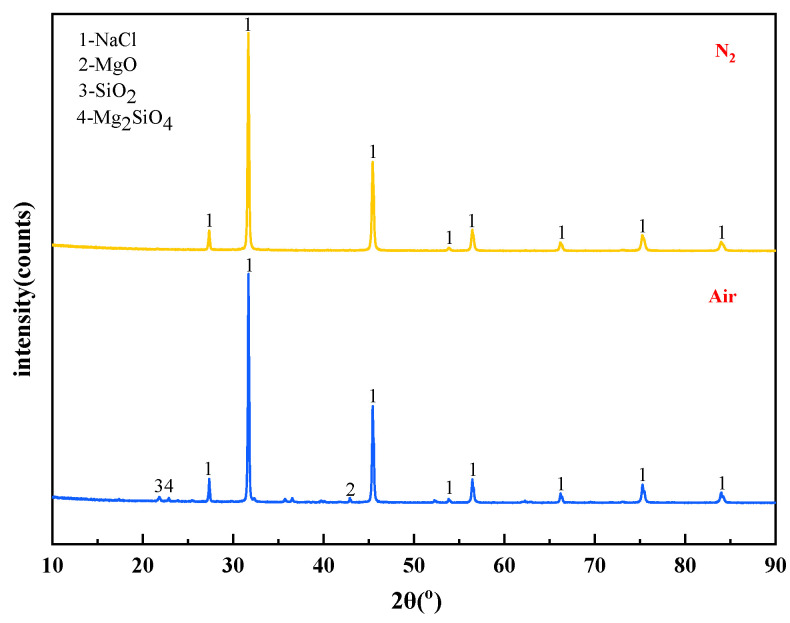
XRD of filtrate obtained with different atmosphere.

The mass content of MgCl_2_ in raw materials is 10%. When the temperature is 1273 K and the molar ratio of added MgCl_2_:Na_2_SiO_3_ is 1:0.5, The X-ray diffraction patterns of the reaction filtrates under different atmospheric conditions are shown in Figure 14.

It can be seen from Figure 14 that the X-ray diffraction peaks of the filtrate under the two atmospheric conditions were similar in position and intensity, and they were all diffraction peaks of NaCl. The content of impurity elements in the filtrate decreased when the reaction atmosphere changed from air to N_2_. It is known that the N_2_ is favourable to obtain high-quality molten NaCl.

**Figure 15:**
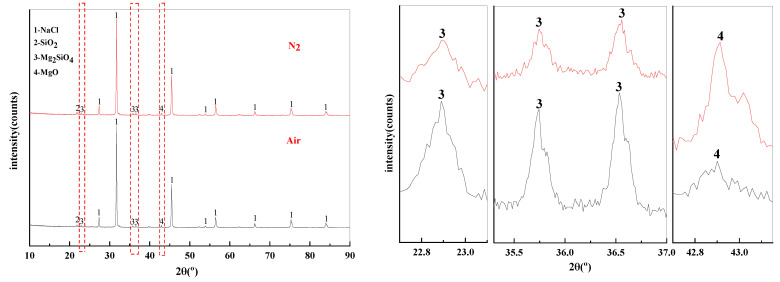
XRD of filter residues obtained with different atmosphere.

The effect of phase transition atmosphere on the phase of filter residues are shown in Figure 15. The influence of the phase transition atmosphere on the phase of filter residues is weak and the type of filter residues phase does not change significantly. The substances contained in the filter residues are NaCl, SiO_2_, Mg_2_SiO_4_, and MgO.

**Figure 16:**
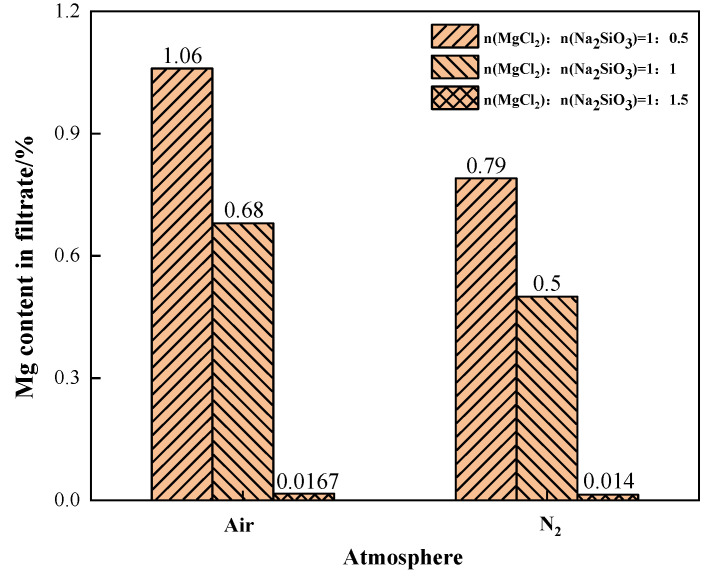
The effect of phase transition atmosphere on the change of Mg content in the filtrate.

The effects of different phase transition atmospheres on the Mg content in the filtrate were investigated under the conditions of additive dosages MgCl_2_:Na_2_SiO_3_ = 1:0.5, 1:1, and 1:1.5, as shown in Figure 16. When N_2_ was the phase transition atmosphere, the Mg content in the filtrate was significantly lower than that in the air atmosphere. This indicates that the N_2_ condition can effectively reduce the content of Mg in the filtrate. When the additive dosage was 1:1.5, the content of impurity Mg in the filtrate was similar and lower in both atmosphere conditions.

**Figure 17:**
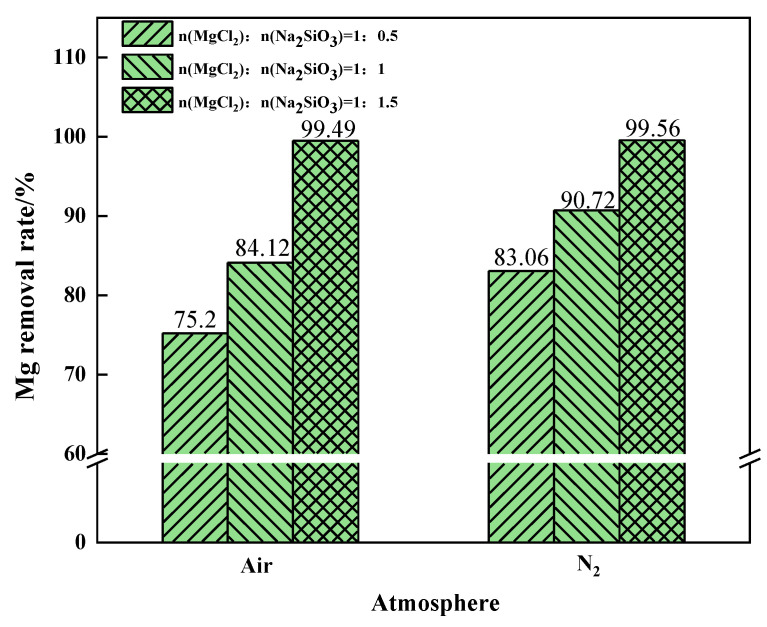
The effect of the phase transition atmosphere on the Mg removal rate.

The Mg removal rate increased with the increase of additive dosage when filtering under air or N_2_ conditions, as shown in Figure 17. When the additive dosage MgCl_2_:Na_2_SiO_3_ was 1:0.5, the increase in Mg removal rate was more obvious after the phase transition experiment under the N_2_ condition. At this time, N_2_ should be selected as the phase transition atmosphere in order to remove as much Mg as possible from the raw material. When the additive dosage MgCl_2_:Na_2_SiO_3_ increased to 1:1.5, the Mg removal rate remained similar under the two atmospheric conditions.

The air atmosphere reprsents the benchmark experimental condition for this experiment. The comparison shows that an oxidising atmosphere inhibits the conversion of magnesium. The inert atmosphere reaction conditions are more conducive to the separation of magnesium. To sum up, while ensuring that Mg in the raw material is removed as far as possible, it is necessary to reduce the influence of the addition on the experiment. Under the same additive dosages conditions, increasing the phase transition temperature is helpful to improve the removal rate of Mg. The N_2_ atmosphere is more suitable for the experiments.

## 5. NaCl Recovery from Molten Salt Chloride Slags

In the previous text, high-temperature phase transition experiments using pure reagent NaCl-MgCl_2_ were performed to simulate the molten salt system. However, the actual molten salt system possesses a more complex composition. In order to put the results into context, the pure reagent NaCl-MgCl_2_-CaCl_2_-C were used to simulate the molten salt system through high-temperature phase transition experiments. At the same time, molten salt chloride slags from Panxi were used for verification experiments.

### 5.1. NaCl Recovery from Molten Salt Chlorinated Synthetic Slags

**Figure 18:**
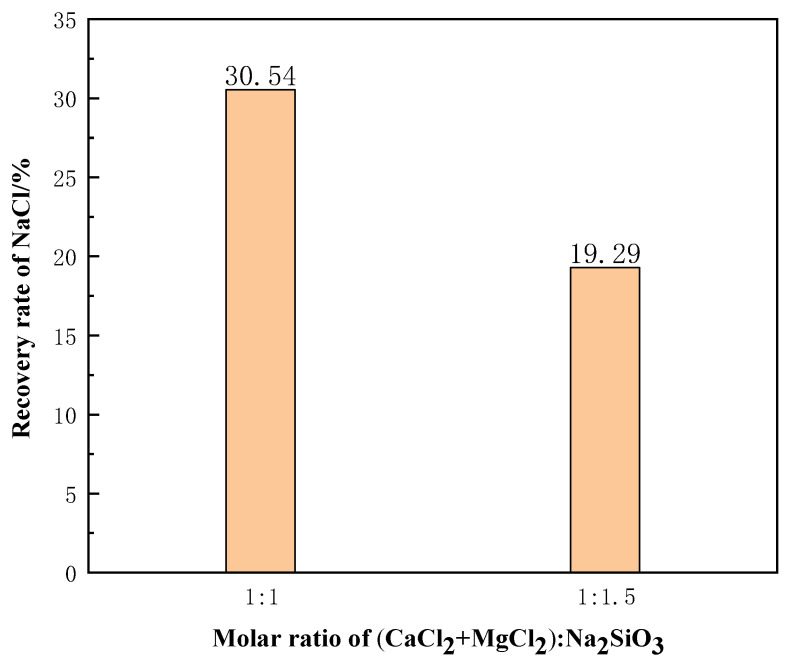
Effect of different additive dosages on the recovery rate of NaCl.

Pure chemical reagents were used to simulate molten salt chloride slags. The molar ratio of NaCl:CaCl_2_:MgCl_2_:C was 75:5:15:5. When the phase transition time was 10 min, the slags were filtered for 90 min at 1273 K. Figure 18 shows the recovery rate of NaCl from the molten salt chlorination synthetic slags with different additive contents. The NaCl recovery rate obtained decreases as the additive content increases. This was due to the high calcium and magnesium content in the molten salt chlorination synthetic slags, and with the increase of additives, more calcium and magnesium silicates were generated. This led to the clogging of the filter and reduction in NaCl recovery rate.

**Figure 19:**
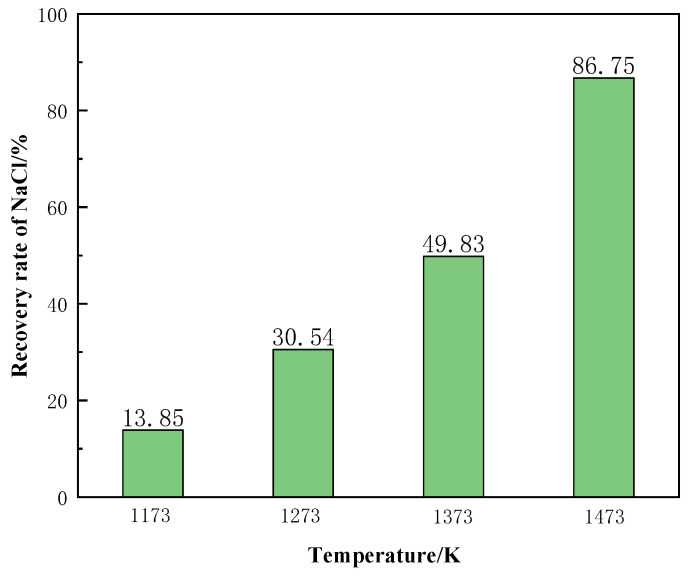
Effect of different temperatures on the recovery rate of NaCl.

Figure 19 shows the recovery of NaCl from the molten salt chlorination synthetic slags at different temperature conditions. The recovery rate of NaCl increased significantly with the increase of temperature. When the temperature was 1473 K, the recovery rate of NaCl reached 86.75%. In summary, the phase transition temperature is the main factor affecting the recovery of NaCl from molten salt chlorination synthetic slags.

### 5.2. NaCl Recovery from Molten Salt Chloride Slags of Panxi

The molten salt chloride slags of Panxi were first roasted at 1123 K for one hour, and then 45 g of the roasted products were weighed for the experiment. When the molar ratio of additive dosage (CaCl_2_+MgCl_2_):Na_2_SiO_3_ was 1:1.5, high-temperature filtration was performed for 90 min at different phase transition temperatures and phase transition times. The experimental results are shown in Table 4.

When the high temperature phase transition was 1373 K for 30 min, the molten salt chloride slags showed an incomplete molten state. At this time, high-temperature filtration was carried out, and the recovery rate of NaCl was extremely small. However, when the temperature reached 1473 K, the recovery rate of NaCl could reach 97.62%. The weight and composition of filtrate in molten salt chlorinated slag system are shown in Table 5. The purity of the recovered NaCl was 99.35 wt%, which met the standards of the molten salt chlorination process.

## 6. Conclusions

High-temperature phase transition recycling of molten salt chloride slags is a clean and efficient method to treat molten salt chloride slags. In this paper, the transition of the Mg-containing phase and its separation from NaCl are systematically investigated, including thermodynamic analysis, phase transition behavior, and Mg removal behavior. When Na_2_SiO_3_ was used as an additive and the temperature exceeded 1073 K, MgCl_2_ could be converted to Mg_2_SiO_4_, which is a solid phase, and NaCl could remain in the liquid phase. XRD analysis showed that the filtrate was mainly in the NaCl phase and that Mg_2_SiO_4_ precipitation was formed. This is the result of the reaction between the addition of Na_2_SiO_3_ and MgCl_2_. The Mg removal rate was 99.56% at 1273 K and N_2_ atmosphere when the MgCl_2_:Na_2_SiO_3_ molar ratio was 1:1.5. The NaCl prepared by this method can be used in molten salt chlorination reactions. Mg_2_SiO_4_ can be used as a construction material. Experiments were conducted to study the NaCl recovery from both synthetic and real molten salt chlorination slag. The high temperature phase transition temperature of molten salt chlorination slag is higher than that of the synthetic slag due to its wide range of components and complex high temperature phase transition reaction. The recovery rate of NaCl from the molten salt chlorination slag was 97.62% and the purity of NaCl obtained was 99.35 wt%, which could be used in the molten salt chlorination process.

## Figures and Tables

**Table 1:** Specifications of the chemicals used in the study.

Name	Chemical Formula	Specific Specifications
Sodium Chloride	NaCl	analysis reagent
Anhydrous magnesium chloride	MgCl_2_	analysis reagent
Anhydrous calcium chloride	CaCl_2_	analysis reagent
Sodium silicate	Na_2_SiO_3_	SiO_2_ 44–47%

**Table 2:** The main chemical composition of the molten salt chloride slag.

Chemical Element	Ca	Mg	Al	Fe	Cl	Na	Mn	Si	Ti
**Content %**	2.76	4.54	2.82	6.46	33.60	9.34	1.24	5.47	7.51

**Table 3:** The parameters in the Equations (1) and (2).

Symbol	Paraphrase
$\eta_{\mathrm{Mg}}$	the mass content of Mg in the filtrate (after separation)
$P_{\mathrm{NaCl}}$	the recovery rate of NaCl
$\omega_{\mathrm{Mg}}^{\prime }$	the mass content of Mg in the filtrate (after separation)
$\omega_{\mathrm{Mg}}$	the total mass content of Mg in the reactants
$\omega_{\mathrm{NaCl}}^{\prime }$	the mass content of NaCl in the filtrate (after separation)
$\omega_{\mathrm{NaCl}}$	the total mass content of NaCl in the reactants
$m_{1}$	the mass of filtrate
$m_{2}$	the mass of NaCl produced by the reaction of Na_2_SiO_3_ with MgCl_2_ (and CaCl_2_)
m	the mass of raw material

**Table 4:** Recovery rate of NaCl at different phase transformation temperatures and times.

Temperatures/K	Times/min	Recovery Rate of NaCl/%
1373	30	33.59
1473	10	97.62

**Table 5:** The filtrate of molten salt chloride slag from Panxi.

Weight of Filtrate/g	Composition of Filtrate/wt%	Recovery Rate of NaCl/%	Mg Removal Rate/%
18.89	NaCl (99.35), MgCl_2_ (0.0063), CaCl_2_ (0.045)	97.62	99.98

## Data Availability

Data is contained within the article.
